# Patient-reported experiences and outcomes of virtual care during COVID-19: a systematic review

**DOI:** 10.1186/s41687-023-00659-8

**Published:** 2023-12-01

**Authors:** Bishnu Bajgain, Sarah Rabi, Sadia Ahmed, Veronika Kiryanova, Paul Fairie, Maria J. Santana

**Affiliations:** 1 Department of Community Health Sciences, University of Calgary, Calgary, AB Canada; 2 Department of Pediatrics, University of Calgary, Calgary, AB Canada; 3 Alberta SPOR SUPPORT Unit, Patient Engagement Team, Calgary, AB Canada; 4 Patient and Community Engagement Research, University of Calgary, Calgary, AB Canada

**Keywords:** Virtual care delivery, Patient-centered care, COVID-19, Patient experience, Patient-reported outcomes, Healthcare service utilization

## Abstract

**Introduction:**

The onset of COVID-19 has caused an international upheaval of traditional in-person approaches to care delivery. Rapid system-level transitions to virtual care provision restrict the ability of healthcare professionals to evaluate care quality from the patient's perspective. This poses challenges to ensuring that patient-centered care is upheld within virtual environments. To address this, the study’s objective was to review how virtual care has impacted patient experiences and outcomes during COVID-19, through the use of patient-reported experience and outcome measures (PREMs and PROMs), respectively.

**Methods:**

A systematic review was conducted in accordance with the Preferred Reporting Items for Systematic Reviews and Meta-Analysis guidelines to evaluate patient responsiveness to virtual care during COVID-19. Using an exhaustive search strategy, relevant peer-reviewed articles published between January 2020 and 2022 were pulled from MEDLINE, CINAHL, EMBASE, and PsychInfo databases. Study quality was independently assessed by two reviewers using the Mixed Methods Appraisal Tool. A patient partner was consulted throughout the study to provide feedback and co-conduct the review.

**Results:**

After removing duplicates, 6048 articles underwent title and abstract review, from which 644 studies were included in the full-text review stage. Following this, 102 articles were included in the study. Studies were published in 20 different countries, were predominantly cross-sectional, and reported on the delivery of virtual care in specialized adult outpatient settings. This review identified 29 validated PREMs and 43 PROMs. Several advantages to virtual care were identified, with patients citing greater convenience, (such as saving travel time and cost, less waiting experienced to see care providers) and increased protection from viral spread. Some studies also reported challenges patients and caregivers faced with virtual care, including feeling rushed during the virtual care appointment, lack of physical contact or examination presenting barriers, difficulty with communicating symptoms, and technology issues.

**Conclusion:**

This review provides supportive evidence of virtual care experiences during the COVID-19 pandemic from patient and caregiver perspectives. This research provides a comprehensive overview of what patient-reported measures can be used to record virtual care quality amid and following the pandemic. Further research into healthcare professionals’ perspectives would offer a supportive lens toward a strong person-centered healthcare system.

**Supplementary Information:**

The online version contains supplementary material available at 10.1186/s41687-023-00659-8.

## Introduction

The SARS2-Coronavirus 2019 (COVID) crisis has severely impacted public health and disrupted the provision of healthcare, including organizing, mobilizing, and deploying extra resources to effectively address emerging needs [1]. For instance, healthcare service delivery has been impacted in numerous ways [2], changing many essential elements vital to providing person-centered care (PCC) [3, 4], and implementing widespread use of virtual care.

Virtual care is defined as any interaction between patients and/or members of their circle of care, occurring remotely, using any forms of communication or information technologies (e.g., phone calls, videoconferences, and secure messages), to facilitate or maximize the quality and effectiveness of patient care [2, 5, 6]. Virtual care can play a vital role in emergencies by supporting healthcare needs remotely [7], streamlining the necessity of healthcare services, conserving medical resources [8], directing the medical supply on the basis of priority [9], and providing telecommunication for visitor-patient interaction[10, 11].

The COVID pandemic resulted in changes to the patient care environment, impacting the delivery of PCC [2–4]. PCC promotes adherence to treatment, improved care, better health outcomes, enhanced relationships between providers and patients, improved perceptions of doctor performance, and patient trust [3]. PCC is advocated by both patients and providers as it supports a higher quality of care [3].

Worldwide, over fifty-eight percent of the countries that experienced service disruption during the pandemic adopted virtual care delivery to continue to meet healthcare needs [12]. Hence, it is crucial to understand the impact of virtual care delivery on patient experiences and outcomes. Additionally, to deliver good patient-centred care, we need to understand what barriers or challenges present and how virtual care can be optimized. Thus, we conducted this systematic review to identify evidence on how virtual care delivery has impacted patient experiences and outcomes, both measured using validated Patient-Reported Experience Measures (PREMs) and Patient-Reported Outcome Measures (PROMs) respectively, during the first 2 years of the COVID pandemic across a spectrum of diseases and healthcare settings.

PROMs are used to assess a patient’s health status at a particular point in time, which can be completed either during an illness or while treating a health condition, or pre-and post-event to measure the impact of an intervention [13]. Capturing patient experiences is an important part of an overall effort to measure health system performance and is integral to delivering patient-centred care. Routinely applying PROMs and PREMs can enhance communication between patients and care providers, inform decisions for value-based healthcare, and improve patient care experiences and outcomes. To achieving health system goals, PROMs and PREMs are increasingly recognized for providing valuable and essential information [13]. With the onset of the COVID-19 pandemic, as the healthcare systems evolve, it becomes increasingly significant to measure healthcare delivery, PREMs, PROMs, and clinical outcomes towards a strong person-centred healthcare system.

## Materials and methods

Based on the exploratory nature of this review and our objective to describe and map the literature guided by our aim outlined above, a systematic review approach was selected. The strength of the systematic review methodology is that it provides a rigorous and transparent approach of mapping the literature to ensure reliable and meaningful results for end-users [14]. Study selection and screening process was performed using the Preferred Reporting Items for Systematic Review and Meta-analysis (PRISMA) methodological frameworks [15]. The PROSPERO registration number is CRD42022306179. Additionally, we engaged a patient research partner with experience accessing virtual care during the COVID-19 pandemic. Our patient research partner was engaged in the conduct of this review (reviewing the study protocol, search strategy, assisting in title and abstract screening, data abstraction, reviewing this manuscript and is a co-author).

### Search strategy and information sources

The preliminary search strategy was developed in collaboration with a research librarian at our University, who also has research expertise in systematic reviews. The search strategy and keywords are presented in Additional file 1. The search strategy combined structure language, keywords, and relevant synonyms. The search terms for each concept were connected through Boolean Operators ‘AND’, while search terms within each concept were combined using ‘OR’. The comprehensive search terms were tailored to each data sources, including MEDLINE, CINAHL, EMBASE, and APA PsycInfo, published from January 2020 until January 2022. To minimize publication bias and missing any relevant literature, we performed an additional search from reference lists of the included studies and grey literature sources, including google scholar and conference proceedings.

### Inclusion and exclusion criteria

*Inclusion criteria*: (1) Population: patient, caregiver, and family member; (2) Intervention: delivery of virtual care during COVID-19; (3) Outcome: virtual care experiences, and outcomes reported by patient/caregiver/family, as well as health utilization outcomes; (4) Study Design: any studies (qualitative, quantitative, and mixed methods); (5) Peer-reviewed studies published only in English language; and (6) Only studies that used validated measures (PROMs and PREMs), as reported by the authors of the included studies.

*Exclusion criteria*: (1) Provider's experience; (2) Use of unvalidated patient-reported measures; (3) Clinical trials (RCT), research protocols, discussion summaries, abstracts and conference posters, systematic reviews, editorials, and letters; (4) Studies that were not in the English language.

### Selection of sources of evidence

References for all included studies were uploaded and managed through Covidence. Titles and abstracts were screened for eligibility by two independent reviewers. Our team of reviewers initially screened 50 references together to ensure consistency between reviewers. For the full-text articles inclusion screening, the first five articles were reviewed by all the reviewers to ensure consistency. After that, each full-text article was reviewed by two independent reviewers. Differences between reviewers were resolved through detailed discussion and consensus or consulting a third reviewer. Differences between reviewers were resolved through detailed discussion and consensus or consulting a third reviewer.

### Data charting process and data items

Two independent reviewers abstracted all relevant data following the full-text screening process for eligibility. A standardized data abstraction form was created to process all data. This form was first piloted by trained reviewers for at least two studies and revised until the authors were satisfied that all relevant data was captured accurately and comprehensively. The following information was extracted from each study for collective evaluation: author, year of publication, country, objectives, study design, patient population, virtual care delivery methods, patient reported experiences, patient reported outcomes, and health utilization outcomes. One reviewer abstracted the data, and the second reviewer checked/verified the abstracted data. Any disagreement in the abstracted data was resolved through discussion and consensus between the two reviewers, or a third reviewer was consulted, if need be. The data items abstracted from each study are presented in Tables 1, 2, and 3.

**Table 1:** Descriptive characteristics of all included articles

Study characteristics	Frequency of articles (n = 102), N(%)
*Study design*	
Cross sectional study	34 (33.3)
Cohort study	31 (30.4)
Qualitative research	13 (12.7)
Mixed methods	12 (11.8)
Case series	3 (2.9)
Other	9 (8.86)
*Healthcare setting*	
Specialized outpatient	80 (78.4)
Acute hospital care	8 (7.8)
Primary care	5 (4.9)
Rehabilitation centre	4 (3.9)
Mental health program	1 (0.98)
Primary and specialized outpatient care	1 (0.98)
Rural care	1 (0.98)
Primary and postnatal care	1 (0.98)
Primary and pharmaceutical care	1 (0.98)
*Age of study population*	
Adult	73 (71.6)
Adult; pediatric	15 (14.7)
Pediatric	11 (10.8)
Not Specified	3 (2.9)
*Mode of virtual care*	
Video call	34 (33.3)
Telephone	22 (21.6)
Remote monitoring	2 (2.0)
Combination (telephone and video)	35 (34.3)
Other	9 (8.2)

**Table 2:** PREMs listed in the included articles [17, 20, 21, 24, 25, 27–32, 38, 40, 44–46, 49–51, 54, 60–64, 66–79]

Author	Study design	Name of PREM	Domains of patient experiences	Who completed?	Summary of findings
Sarre 2021 [40]	Cross-sectional	Care Quality Commission's Maternity Services Survey 2019	(i) The start of care in your pregnancy, (ii) antenatal check-ups, (iii) during your pregnancy, (iv) labour and birth, (v) staff caring for you, (vi) care in hospital after the birth, (vii) feeding your baby, and (viii) care at home after the birth	Adult patient	Most patients felt listened to during their virtual care appointments, although 49% did provide at least one challenge they had trying to access virtual care
Assenza et al. 2020 [61] Wyler et al. 2021 [62]	Cross sectional Mixed methods	Client Satisfaction Questionnaire (CSQ)	N/A	Adult patient or proxy [61] Adult patient [62]	Relatively high satisfaction was reported for both virtual care programs
Zimmerman et al. 2022 [63]	Cohort	Clinically Useful Patient Satisfaction Scale (CUPSS)	(i) Clinician's attitude and behavior, (ii) office environment and staff, and (iii) overall satisfaction	Adult patient	Satisfaction with treatment was similar across in-person and virtual care
Pooni et al. 2021 [64] Schumm et al. 2021 [65]	Cross sectional	Communication Assessment Tool (CAT)	(i) Physician-oriented experience, (ii) staff-oriented experience	Pediatric patient [64] Adult patient [65]	Pooni et al. reported that satisfaction scores were highest for domains related to the patient-physician relationship (treated with respect, showing care and concern) In Schumm et al.’s article, top-box scores were lower for telemedicine patients versus in-person for ‘the doctor spent the right amount of time with me’, doctor encouraged me to ask questions” and “the doctor checked to be sure I understood everything”
Santoro et al. [50] Meno et al. [54] Schumm et al. [65] Sharma et al. [66] Shaverdian et al. [67]	Cross sectional	Consumer Assessment of Healthcare Providers and Systems—Clinician and Group Survey (CG-CAHPS	(i) Access to care, (ii) doctor communication, and (iii) courteous/helpful staff	Proxy [50], adult patient [50, 54, 65–67]	In Santoro et al., caregivers found some features of virtual visit to be better (convenient time, childcare or eldercare arrangements, travel, coordination, and wait time). In the other studies as well, virtual care was believed to be comparable if not better than in-person care. However, personal connection with their provider was higher for in-person visits
Kludacz-Alessandri et al. [20]	Cross-sectional	General Practice Assessment Survey (GPAS)	(i) Access, (ii) technical care, (iii) communication, (iv) interpersonal care, (v) trust, (vi) knowledge of patient, (vii) nursing care, (viii) receptionists, (ix) continuity of care, (x) referral, (xi) coordination of care, (xii) patient recommendation of GP, and (xiii) overall satisfaction	Adult patient	~ 55% of patients sampled stated that the quality of in-person and virtual care were comparable. Benefits of virtual care related to cost and time, while issues centered on difficulty accessing electronic medical records
Darr et al. [25]	Cohort	General Medical Council (GMC) Patient Questionnaire	N/A	Pediatric patient or proxy	Almost all patients responded agree or strongly agree in the domains of the patient-physician relationship, privacy, and trust for virtual consultations
Darr et al. [25]	Cohort	Telemedicine Satisfaction and Usefulness Questionnaire (TSUQ)	(i) Usefulness and (2) satisfaction	Pediatric patient or proxy	Patient satisfaction with virtual care was high in the domains of the patient-physician relationship, privacy, and trust
Horgan et al. [31]	Cohort	Generic Medical Interview Satisfaction Scale (G-MISS)	(i) Relief, (ii) communication, and (iii) compliance	Adult patient	Satisfaction for virtual care was high, with ~ 80% stating they would have a telephone consultation again
Dratch et al. [23]	Cohort	Genetic Counseling Satisfaction Scale (GCSS) [23]	N/A	Adult patient	Most patients (73%) felt virtual care improved access to services and voiced a preference for a combination of in-person and virtual care in the future
Wee et al. [68]	Cross-sectional	Groups Health Association of America Patient Satisfaction Survey	N/A	Adult patient	No significant difference in patient satisfaction was measured between, in-person, direct access, or virtual care
Piro et al. [69]	Cohort	Home Monitoring Acceptance and Satisfaction Questionnaire (HoMASQ)	(i) Relationship with their healthcare provider, (ii) ease of use of home monitoring technology, (iii) related psychological aspects, (iv) implications on general health, and (v) overall satisfaction	Adult patient	No significant difference between in-person and virtual (home) care satisfaction were provided; however, self-reported anxiety during virtual care was lower than in-person
Quinn et al. [21]	Cross sectional	Telephone Clinic Care survey	N/A	Adult patient	86% of women described their virtual consultation as “good” or “very good” with 25% preferring virtual over in-person care
Bartoletta et al. [30]	Cohort	Press Ganey Medical Practice Telemedicine Survey	(i) Accessibility to virtual clinical encounter, (ii) care provider, (iii) telemedicine technology, and (iv) overall assessment of telemedicine visit	Adult patient	Most patients responded that arranging and connecting to a telemedicine visit, talking with the provider over a video connection, and having the provider understand the clinical problem were “very good”
Bartoletta et al. [30] Porche et al. [71] Richards et al. [70]	Cohort Cross sectional	Press Ganey Outpatient Medical Practice Survey (PGOMPS) 2023-11-17 7:33:00 PM	(i) Access, (ii) moving through your visit, (iii) nurse/assistant, (iv) care provider, (v) personal issues, (vi) overall assessment, and (vii) telemedicine technology (added March 2020)	Adult patient	Satisfaction across all patient groups receiving virtual care was high, with improvement suggestions targeting technological challenges. [30, 70] No significant difference in satisfaction was measured between virtual and in-person care. [71]
Alshareef et al. [51]	Cross sectional	Questionnaire for Assessing Patient Satisfaction with Video Teleconsultation	(i) Equipment/technical issues, (ii) communication and rapport, (iii) clinical assessment, and (iv) program evaluation	Adult patient	Thirty-seven respondents (90.2%) agreed that telemedicine is cost and time efficient compared to conventional in-office visits. Of the 41 respondents, 35 (85.4%) patients thought it was easy to gain access to specialist care by telemedicine
Pooni et al. [64]	Cross sectional	RAND Visit-Specific Satisfaction Instrument (VSQ-9)	N/A	Pediatric patient	Patient satisfaction was related most closely with the caregiver’s inter-personal and technical skills. The lowest rated scores addressed patient wait times and challenges accessing the office by phone
Zimmerman et al. 2020[44]	Cross sectional	Service User Technology Acceptability Questionnaire (SUTAQ)	(i) Enhanced care, (ii) increased accessibility, (iii) privacy and discomfort, (iv) care personnel concern, (v) kit as substitution and (vi) satisfaction	Adult patient	Most patients stated that virtual care saved them time, increased care accessibility, improved their health, and facilitated improved monitoring of their symptoms
Wyler et al. [62]	Mixed Methods	Session Evaluation Questionnaire (SEQ)	(i) Session evaluation and (ii) post-session mood	Adult patient	Patient satisfaction with in-person and virtual care were not significantly different, however, patients and therapists did state the virtual care produced more superficial dialogue
Birkhoff et al. [27]	Mixed Methods	System Usability Scale (SUS)	N/A	Adult patient	50% of patients felt that in-person and virtual nursing visits provided comparable care. Patients that did not prefer virtual care mentioned difficulties navigating technology or taking their own vitals
Mojdehbakhsh et al. [17] Darr et al. [25] Edge et al. [28] Efthymiadis et al. [46] Chen et al. [72]	Cohort Cross-sectional	Telehealth Satisfaction Scale (TeSS)	N/A	Adult patient [17, 28, 46], pediatric patient or proxy (25), adult or pediatric patient or proxy [72]	Satisfaction across all patient groups receiving virtual care was high, however, Edge et al. reported lower scores regarding the psychological and emotional support the patients’ received through virtual care
Peahl et al. [19] Dratch et al. [24] Darr et al. [25] Adamou et al. [33] Triantafillou et al. [35] Waqar-Cowles et al. [39] Fung et al.[45] Galaviz et al. [74] Park et al. [52] Finn et al. [73] Al-Sharif et al. [58]	cohort Cross sectional Mixed methods	Telehealth Usability Questionnaire (TUQ)	(i) Usefulness, (ii) ease of use, (iii) effectiveness (interface and interaction quality), (iv) reliability, and (v) satisfaction	Proxy [45, 74] adult patient [19, 24, 33, 35, 73] adult or pediatric patient [52, 58] adult patient or proxy [39] pediatric patient or proxy [25]	When experiential domains are assessed individually, satisfaction is reported to be relatively high, however, when virtual is directly compared to in-person care, satisfaction scores showed a significant drop in score. Triantafillou et al. [35], reported lower satisfaction levels addressing the patient-physician relationship
Waqar-Cowles et al. [39]	Cross sectional	Telemedicine practice in neurosurgery during COVID-19 era: an anonymized patient survey	N/A	Adult patient	~ 97% of patients felt that their virtual care was beneficial in the context of COVID-19, with 33% of patients stating a preference for virtual over in-person care. The most common suggestion for the program was to implement video-based consults
Nair et al. [32] Rush et al. [78] Wright et al. [75] Pareyson et al. [77] Pinar et al. [76] Banks et al. [49]	Cross sectional Cohort Mixed-methods	Telemedicine Satisfaction Questionnaire (TSQ)	(i) Quality of care provided, (ii) similarity to face-to-face encounter, and (iii) perception of the interaction	Adult patient [32, 49, 75, 76, 78] adult patient or proxy [77]	Satisfaction across all patient groups was relatively high, however, preference for virtual care was particularly associated with difficulty accessing in-person services and current disease stability
Ruelos et al. [23]	Cross sectional	Telehealth Satisfaction Questionnaire	Communication; convenience; privacy; other: ability to save time, trust of the telehealth system, the system is easy to learn, lack of physical contact, ability to meet patient's needs	Adult	Most patients favored telehealth because of its ease of use, convenience, and ability to save time
Murthy et al. [79]	Cross sectional	Your Virtual Appointment: Your Experience	N/A	Adult patient	Preference for in-person (14%) relative to virtual care (19%) was mixed, with technological difficulties and clinical limitations referenced for virtual care hesitancy
Watson et al. [38]	Mixed methods	Your Voice Matters (YVM)	(i) Appoint arrival, (ii) appoint duration, and (iii) leaving the appointment	Adult patient	Measured levels of satisfaction were significantly lower among patient receiving treatment relative to those undergoing follow-up
Raheja et al. [29]	Cross sectional	Patient satisfaction with telemedicine services survey	Communication; information	Adult patient	The majority of patients (either agreed/strongly agreed that the time allotted for their queries during teleconsults was sufficient
Imlach et al. [26]	Mixed Methods	Primary Care Patient Experience Survey	Respect; communication; convenience; Other: spend enough time, need to be seen in-person, relationships, technological barriers, views on values, patient preferences	Adult patient	Many respondents mentioned the convenience of telehealth consultations, in terms of saving time and money, and reducing stress, travel, employment disruption and exposure to infection (with COVID-19 and other pathogens)

Included surveys: [80–86]

**Table 3:** PROMs listed in the included articles [63, 69, 90–109]

Author	Study design	Disease/condition specific group	Name of PROM	Summary of findings
Riegler et al. [98]	Cohort	Mental Health	Center for Epidemiological Studies Depression Scale (CES-D)	Veterans reported significant reduction in symptoms of depressions after the telepsychotherapy parenting skills intervention
			Eyberg Child Behavior Inventory	Frequency of clinically significant scores pre- and post-treatment were significantly different
Levinson et al. [103]	Cohort		Beck Depression Inventory II	Changes in BDI scores were not significantly different between in-person and virtual groups
			The Frost Multidimensional Perfectionism Scale (FMPS)	Changes in FMPS scores were not significantly different between in-person and virtual groups (pre to post treatment)
Bulkes et al. [108]	Cohort		Quick Inventory of Depression Symptomology-Self Report (QIDS-SR)	Admission and discharge QIDS-SR scores for the in-person and virtual care groups were not significantly different
Zimmermann et al. [63]	Cross sectional		Remission from Depression Questionnaire (RDQ)	Significant improvements in RDQ scores between time points were recorded for virtual relative in in-person care
Raykos et al. [92]	Case series		PROMIS-Anxiety and Depression scales, Short Form 8a	Statistically significant improvements in PROMIS scores were reported as therapy progressed
			Eating Disorders-15 Questionnaire (ED-15)	Statistically significant improvements in ED-15 scores were reported as therapy progressed
Raykos et al. [92] Levinson et al. [103] Steiger et al. [104]	Case series Cohort		Eating Disorder Examination Question Version 4.0 (EDE-Q-IV)	Levinson and Steiger et al. reported that changes in EDE-Q-IV scores were not significantly different between in-person and virtual groups. Raykos et al., found statistically significant improvements in EDE-Q-IV scoring as therapy progressed
Rezich et al. [109]	Cross sectional		Hospital Anxiety and Depression Scale (HADS)	Patient responsiveness to telehealth was unconnected to their respective HADS score
Piro et al. [69] Craig et al. [90] Steiger et al. [104] Graziano et al. [99]	Cohort Experimental		Generalized Anxiety Disorder (GAD-7)	Craig et al. [90], reported no significant difference in GAD-7 scores between satisfied and unsatisfied patients. Graziano et al. [99], showed significant reductions in depression at the two time points. Lastly, Steiger et al. [104], calculated negligible interactions between the mode of care delivery and GAD scoring
van Agteren et al. [100]	Experimental		Mental Health Continuum Short Form (MHC-SF)	Participants reported mental well-being improvements before and after exposure to virtual care
Ahmad et al. [91]	Cohort	Gastrointestinal	Baylor Continence Score (BCS)	BCS scores significantly improved following the virtual Bowel Movement Program
			Cleveland Clinic Constipation Score (CCCS)	CCCS scores did not significantly improve following the virtual Bowel Movement Program
			Vancouver Symptom Score for Dysfunctional Elimination Syndrome (VSS)	Significant improvements in VSS scores were reported at the 1-month and 3-month follow-up time points
Sabbagh et al.[102]	Cohort	Musculoskeletal	American Shoulder and Elbow Surgeons (ASES) Standardized Shoulder Assessment Form	No significant difference in outcomes were reported between the virtual and in-person care groups
Ganderton et al. [94]	Longitudinal (pre-post intervention)		Melbourne Instability Shoulder Scale (MISS)	Significant and clinically relevant improvements in MISS scores were reported at follow-up
			Short form Orebro Musculoskeletal Pain Questionnaire (ÖMSPQ)	Significant improvements in patient scores were reported at all follow-up meetings
			Shoulder Instability Index (WOSI)	Excluding the physical subsection of the PROM, patients reported significant improvements in their WOSI scores
			Tampa Scale for Kinesiophobia	The scale reported clinically significant reductions in pain-related fear at the 6-week and 12-week time points
Alsobayel et al. [95]	Cross sectional		Musculoskeletal Health Questionnaire (MSK-HQ)	Significant improvements in the MSK-HQ were recorded between baseline and follow-up
			Pain Self-Efficacy Questionnaire	Significant improvements in the Pain Self-Efficacy Questionnaire were recorded between baseline and follow-up
Wu et al. [106]	Cohort		Functional Assessment of Chronic Illness Therapy (FACIT)—Fatigue Scale	A significant increase in FACIT scores were reported after treatment
Craig et al. [90]	Cohort		Neck Disability Index (NDI)	No significant difference in NDI scores were reported between satisfied and unsatisfied patients
			Oswestry Disability Index	No significant difference in Disability Index scores were reported between satisfied and unsatisfied patients
Corona et al. [107]	Cohort	Early Childhood Development	MacArthur-Bates Communicative Development Inventory (MCDI)	No statistically significant findings were reported between the in-person and virtual treatment groups
Lai et al. [96]	Experimental	Neurological	Quality of Life in Alzheimer's Disease (QoL-AD)	Significant improvements in QoL-AD scoring were reported for video calling relatively in audio-only meetings
			Revised Memory and Behavior Problem Checklist (RMBPC)	No significant improvements in RMBPC scoring were reported for video calling relatively in audio-only meetings
Daswani et al. [97]	Case series	Respiratory	St George's Respiratory Questionnaire (SGRQ)	An average SGRQ improvement of 71% was reported by patients
*Generic PROMs*				
Raykos et al. [92]	Case series	N/A	Clinical Impairment Assessment (CIA)	Large decreases in patient-reported impairment was recorded
Thesenvitz et al. [105]	Cohort		EuroQol Five Dimension—Five Level (EQ-5D-5L)	Reported no statistically significant difference in scores between baseline and follow-up
Sabbagh et al. [102] Wu et al. [106]	Cohort		EuroQol Five Dimension—Three Level (EQ-5D-3L)	Wu et al., reported no significant difference in patient scores before and after treatment. Sabbagh et al., found no significant difference in scoring between in-person and virtual treatment groups
Lotan et al. [93]	Case series		Goal Attainment Scale (GAS)	Improvements in patient outcomes were reported in those receiving virtual care
Craig et al.[90] Steiger et al. [104] Graziano et al. [99]	Cohort Experimental		Patient Health Questionnaire (PHQ-9)	Craig et al., reported no significant difference in PHQ-9 scores between satisfied and unsatisfied patients. Graziano et al., showed significant reductions in depression at the two time points. Lastly, Steiger et al., calculated negligible interactions between the mode of care delivery and PHQ-9 scoring
Alsobayel et al. [95]	Cross sectional		Patient-Specific Functional Scale	Significant improvements in the Patient-Specific Functional Scale were recorded between baseline and follow-up
Ahmad et al. [91]	Cohort		Pediatric Quality of Life Inventory (PedsQL)	No statistically or clinically significant improvements in PedsQL scores were reported
Bulkes et al. [108]	Cohort		Quality of Life Enjoyment and Satisfaction Questionnaire—Short Form (Q-LES-Q)	Admission and discharge Q-LES-Q scores for the in-person and virtual care groups were not significantly different
van Agteren et al. [100]	Experimental		Satisfaction with Life Scale (SWLS)	Participants reported in increase in mental well-being before and after exposure to virtual care
Bernocchi et al. [101] Sabbagh et al. [102]	Cohort		Short form (SF-12) Quality of Life Questionnaire	Sabbagh et al., found no significant difference in scoring between in-person and virtual treatment groups. Bernocchi et al., reported a significant improvement in the mental component but not the physical component of the survey
Lai et al. [96]	Experimental		Short Form 36 Version 2 (SF-36v2) Quality of Life Questionnaire	Significant improvements in SF-36v2 scoring were reported for video calling relatively in audio-only meetings
			Zarit Burden Interview Scale (ZBI)	Significant improvements in ZBI scoring were reported for video calling relatively in audio-only meetings
Riegler et al. [98]	Cohort		Strengths and Difficulties Questionnaire (SDQ)	Statistically significant improvements in the SDQ were reported pre- and post-treatment
Craig et al. [90]	Cohort		Visual Analog Scale (VAS)	No significant difference in VAS scores were reported between satisfied and unsatisfied patients

Included surveys: [38]

### Quality assessment

The Mixed Methods Appraisal Tool (MMAT) Version 2018 was used to assess the quality of all included studies [16]. Using the Mixed-Methods Assessment Tool (MMAT), the included articles demonstrated a relatively high degree of transparency in the presentation of their methods and results. Study quality did not dictate article exclusion from the review and, as such, these articles were still included in data extraction and analysis. Please note as well that using the MMAT questions to generate an overall score or rating of the articles is discouraged by the creators of the tool. For a more thorough overview of included study quality, please refer to the Additional file 2.

### Data analysis

A careful assessment of data and analysis from all included studies was performed to establish and validate any conclusions regarding virtual care experiences during COVID-19. Bibliographic data, the population, and the setting for included studies are summarized using descriptive statistics in Table 1. The patient reported experiences, outcomes, and utilizations are synthesized in Tables 2 and 3. We summarized the results of the qualitative data by patient experience domains, guided by the qualitative software analysis, NVivo.

## Results

**Fig. 1:**
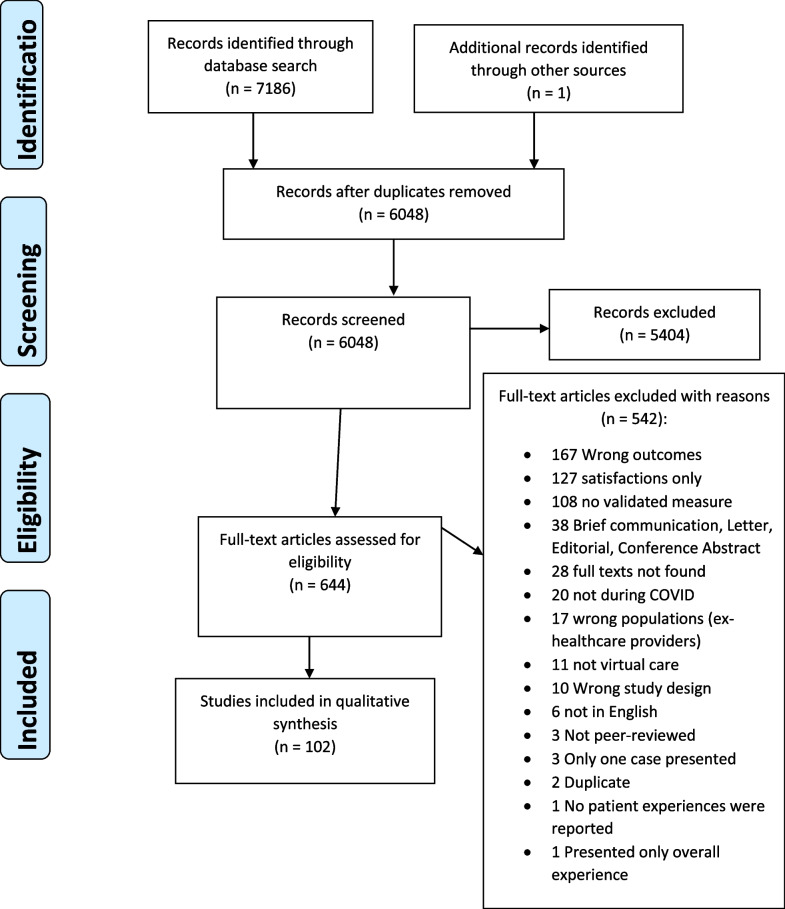
PRISMA for flow chart of the literature review and article identification process

After excluding duplicates, our search captured 6048 records for the title and abstract review. After title and abstract screening, a total of 644 peer-reviewed articles were assessed for full text review. Then, after full text review, we included 102 articles in this review (Fig. 1). Reasons for exclusion included articles reporting wrong outcomes, assessing satisfaction, not validated PROMs/PREMs, full-text unavailable, and wrong timing—not during COVID-19.

**Fig. 2:**
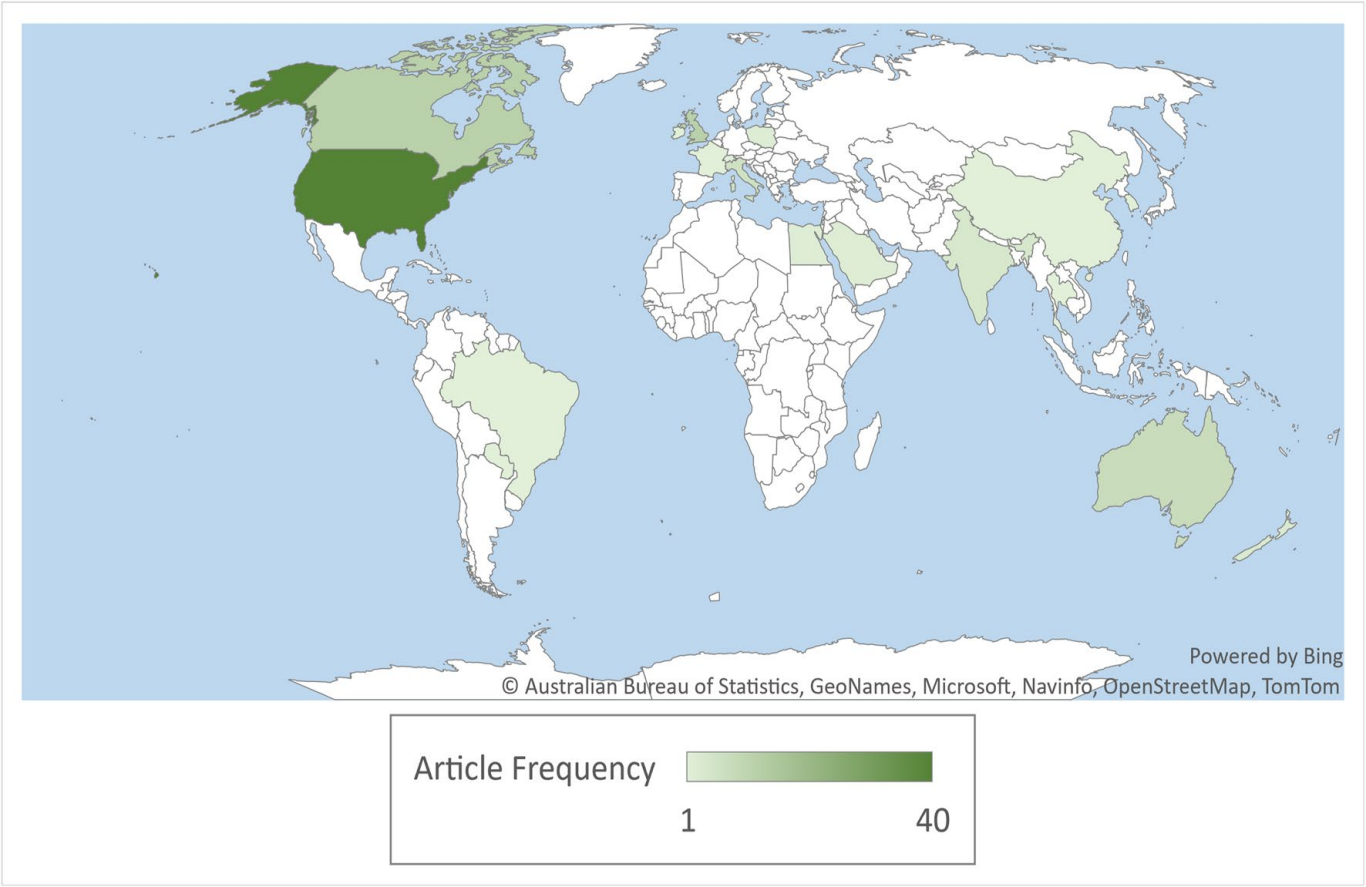
World map displaying the geographic origin of the articles included in the systematic review

Table 1 depicts the descriptive summary of included articles. Most articles (39.2%) were published in the USA, followed by Canada (12.7%), United Kingdom (11.8%), Australia (7.8%), Italy (5.9%), and India (3.9%) (Fig. 2). Studies were of cross-sectional design (33.3%), cohort (30.4%), qualitative (12.7%), mixed methods research (11.8%), case series (2.9%), non-randomized experimental (2%), and other study designs (6.86%). In regard to the quality of the included studies, most articles addressed all (n = 41) or four out of five (n = 42) of the quality criteria listed in the assessment tool. While 19 articles fell below this quality threshold.

Most studies reported virtual care delivered in the specialized outpatient setting (78.4%) during COVID-19. Some studies reported virtual care delivery in acute hospital care settings (7.8%), primary care (4.9%), and in rehabilitation centers (3.9%). Studies reported delivery of virtual care via telephone and video (34.3%), video only (33.3%), telephone only (21.6%), remote monitoring (2%), and other (8.2%). Most studies explored virtual care delivery for adults (71.6%), some reporting both adult and pediatric populations (14.7%), and few articles that reported delivery of virtual care for pediatric populations (10.8%). Most virtual care delivery was in the year 2020 (90.2%).

### Identification of Patient-Reported Experiences Measures (PREMs)

Table 2 highlights the 29 validated Patient-Reported Experience Measures (PREMs) identified in the review from 47 articles that evaluated the patient experience of receiving virtual care during COVID-19. Some articles used more than one PREM. Most of these measures were completed by adult patients. Common measures included The Telehealth Usability Questionnaire (reported by 11 studies), Telemedicine Satisfaction Questionnaire (reported by 6 studies), Telehealth Satisfaction Scale (TeSS) (reported by 5 studies), and Consumer Assessment of Healthcare Providers and Systems—Clinician and Group Survey (CG-CAHPS) (reported by 5 studies). Common domains associated with these measures included access to care, communication, and domains specific to the clinician's attitude and behavior.

### Benefits of virtual care delivery

Findings from patient experience measures (PREMs) highlight positive responses on virtual care from patients in various domains. Some examples include feeling comfortable in receiving care virtually (e.g. due to privacy) (n = 8, 17%) [17–25], feeling safe against COVID-19 (n = 5, 11%) [18, 21, 26–29], communication with healthcare providers (n = 31, 66%) [17, [17, 20–24, 30–43], the convenience of virtual care and saving time (n = 24, 51%) (e.g. minimizing barriers such as transportation, traffic, cost of gas and parking, and associated anxiety) [20–23, 26, 28–33, 35, 37–39, 43–55], access to care (n = 9, 19%) [19, 22, 24, 28, 35, 38, 43–45, 51, 56, 57], patient engagement in care (n = 4, 9%) [36, 37, 44], comfort in the technology/telehealth system (n = 17, 36%) [27, 28, 35, 51], and not experiencing wait time delays in seeing their healthcare providers (n = 8, 17%) [22, 28, 46, 50, 54].

In the studies that included qualitative findings, we get an in-depth understanding of the experiences of patients and caregivers with virtual care delivery during COVID-19. For instance, in the study by Al-Sharif et al.[58], they found convenience and safety to be two major advantages to virtual care delivery, especially with the high risk of getting infected with COVID-19. Juarez-Reyes et al.[59] found patient participants expressed gratitude for continued mental health support, and being able to still be a part of virtual group sessions.

### Barriers with virtual care

Some studies that used PREMs and also qualitative interviews reported challenges patients and caregivers faced with virtual care, such as feeling rushed during the virtual appointment (2% of PREMs articles) [26, 38, 48], lack of physical contact with the healthcare provider for physical examinations (15% of PREMs articles) [21, 23, 26, 35, 58, 87, 88], technical challenges (2% of PREMs articles) [26, 29, 35, 37, 43, 48, 56, 77], a preference for in-person care delivery (8.5% of PREMs articles) (e.g. due to the lack of personal connection with healthcare provider online) [21, 23, 28, 38, 41, 54, 88, 89], and difficulty with communicating symptoms or asking all of their questions (6% of PREMs articles) [29, 87, 90]. For instance, the study by Gibbs et al. [48] found that adult clients undergoing assessment for autism were concerned about communication difficulties in the online environment, especially with using and reading body language and feeling self-conscious about seeing themselves on screen. Adult clients and parents/caregivers were also concerned with clinicians possibly missing certain subtle behaviors not apparent on screen [47]. The study by Stirling Cameron et al. [42] found telehealth appointments to be challenging for Syrian refugee women who used interpreters for their appointments. The women expressed disappointment with back-and-forth telephone calls, and not being able to effectively communicate with their primary care providers [42].

### Identification of Patient-Reported Outcome Measures (PROMs)

We identified 43 validated Patient-Reported Outcome Measures (PROMs) that assessed patient health status during the COVID-19 pandemic (Table 3). The Generalized Anxiety Disorder (GAD-7) (used in 4 studies), Eating Disorder Examination Question Version 4.0 (EDE-Q-IV) (used in 3 studies), and Patient Health Questionnaire (PHQ-9) (used in 3 studies) were the only measures reported in more than one study. All other studies utilized various PROMs. Specific PROMs were grouped by disease/condition such as PROMs for Mental Health (n = 12, 28%), Gastrointestinal (n = 2, 7%), Musculoskeletal (n = 10, 23%), Early Childhood Development (n = 1, 2%), Neurological (n = 2, 5%), and Respiratory (n = 1, 2%). Additionally, fourteen generic PROMs were identified such as the Short Form (SF-12) Quality of Life Questionnaire, Patient Health Questionnaire (PHQ-9), and the Pediatric Quality of Life Inventory (PedsQL). These studies administered PROMs following virtual care delivery. Some studies found that patients had improvements in quality of life and reduction in symptoms [91–98], such as improvements in mental health and wellbeing [63, 92, 96, 99–101]. However, some studies also reported no significant differences in PROMs scores before and after virtual care or between different treatment groups (in-person care vs virtual care) [90, 91, 96, 102–108]. A summary of the findings from the studies is included in Table 3.

### Impact of virtual care delivery on healthcare use

Eleven studies evaluated the impact of virtual care delivery on healthcare use [105, 108, 110–118]. Nascimento et al. [110] evaluated the impact of telemedicine on visits to emergency departments and hospital admissions during the pandemic in Brazil. They found rates of ED visits and hospital admissions were respectively, 17.3% and 2.3% for patients who attended at least one teleconsultation. Kesavadev et al. [111] reported successful prevention of hospitalization for nearly all patients in a virtual in-patient program. In the study by Thesenvitz et al.[105], patients reported less use of services such as Alberta’s Health Link advice line, emergency department visits, and visits with family physicians.

## Discussion

During the COVID-19 pandemic, infection control efforts have necessitated the reduction of in-person clinical visits and routine procedures leading to provider- and system-level changes in the delivery of PCC. This change might have altered patient experiences with their care, and measuring patient experiences becomes increasingly significant toward a strong person-centered healthcare system. In this review, we provide an overview of the PREMs and PROMs that have been utilized to assess patient experiences with virtual care and patient-reported outcomes during the COVID-19 pandemic. Following initial screening and full-text review, 102 articles were included in this study. These studies demonstrated large heterogeneity in study design, population of interest, and virtual care modality. Most articles targeted the delivery of virtual care in specialized outpatient settings (78.4%), including fields such as oncology, dementia, neurology, urology, dermatology, and psychiatry. Studies also primarily assessed adult responses to virtual care delivery (71.6%), with far fewer studies exclusively assessing the perspectives of pediatric patients (10.8%). We also found that a relatively even number of studies assessed patient experiences with virtual care delivery via videoconferencing (33.3%), telephone calls (21.6%), or a combination of both (34.3%). While prior systematic reviews have examined PREM and PROM utilization in various in-person care settings, this study is distinct in its focus on studies that used patient-reported measures to gauge patient experiences to virtual care during the pandemic.

The sheer number of articles (N = 102) included in this review highlight the breadth of information available on patient-reported measures that were used during the virtual care provision, as well as the adaptability of international health systems. This also provides evidence of the importance healthcare professionals ascribe to amplifying the patient voice. Despite this, review findings also show increased investment in specific patient populations, leading to the potential absence of other patient groups.

One specific group that was underrepresented in this review was pediatric patients, as we found a limited number of studies conducted in this population (10.8% of studies were pediatric focused). The lack of research into the experiences and outcomes of pediatric patients receiving virtual care signifies a gap in knowledge that could provide incredibly useful insight into pediatric care provision. Santoro et al., [50] discuss the foreseeable benefits of virtual care for pediatric patients, highlighting the involvement of one or more caregivers in the transportation and supervision of pediatric patients during in-person visits in 2021. From the pediatric studies in this review, patient caregivers discussed the convenience and cost-benefits of virtual care [18, 50, 107].

A second underrepresented patient population in this review are primary (i.e., general) care recipients. Even though primary care serves as the first interaction many patients have with the healthcare system, patient experience in primary care was only assessed in 4.9% of the articles pulled. Not capturing patient perspectives on virtual primary care delivery could significantly impact other healthcare areas by restricting the ability of general practitioners to communicate, treat, and refer patients to specialists effectively.

Another concern with virtual care provision, irrespective of the patient population being researched, is acknowledging the patients who were unable to access virtual care. Virtual care has been shown to exacerbate health inequities, creating what has been termed the “digital divide” whereby health information technology and virtual care disproportionately exclude already marginalized populations from accessing care [53, 119]. This is of particular concern during the COVID-19 pandemic, as rapid transitions from in-person to virtual care have primarily been implemented using a health systems perspective with limited consideration for diverse patient partnerships [120, 121]. The potential bottlenecking of the types of patients included in this review should therefore be needed, with greater efforts placed on broadening and adapting virtual care efforts to better suit the care needs of all patients in future research.

The patient-reported measures identified in the review often addressed care accessibility, patient-care team communication, and clinician attitudes and behavior with patients during virtual care. Several advantages to virtual care were identified, with patients citing greater convenience and increased protection from viral spread. Other literature supports these findings, explaining the potential of virtual care to alleviate barriers to care in rural and geographically isolated communities [122, 123]. Buyting et al. [124], discusses the benefits of virtual care in rural settings when a priori work is done to ensure all interventions are appropriate to the population of interest. Greater ease of access to care was also evaluated by Darr et al. [25], who identified a correlation between virtual care provision and a reduction in non-attendance rates. This also highlights the potential economic benefits of virtual care, as non-attendance rates are closely linked to increased healthcare utilization [25, 125]. In addition to virtual care's advantages, patients also mentioned various challenges. Barriers to virtual care included difficulty navigating online platforms, a need for greater technical support or educational materials, and the lack of physical interactions with healthcare providers. Edge et al. [28], reported that some patients felt they received worsened psychological support through virtual care and experienced greater difficulty understanding the clinical information shared by their healthcare provider. In response to this, 1 in 5 patients were hesitant to use virtual care in the future [28].

Virtual care is associated with various benefits and challenges, offering increased access to care during times of public isolation but also restricting care to populations experiencing social marginalization or with limited technological infrastructure [126]. The polarity of responses to virtual care raises the question of whether this mode of care will continue beyond the pandemic or if care will largely return to in-person once safe to do so. While a large proportion of patients included in the review mainly expressed positive reactions to virtual care, with some stating a preference for virtual care over in-person visits, consideration should also be applied to those not heard in these studies. Therefore, if this were to continue long-term, greater attention needs to be directed toward making technology a facilitator instead of a barrier to care access. Perhaps the most promising approach to virtual care in the future is implementing specialty-specific triage practices to provide patients with the most appropriate care. Other researchers have proposed this, promoting the benefits of triaging for better allocation of resources, assessment of disease acuity, and accommodation to various social factors [127, 128].

Irrespective of the degree to which virtual care is used in the future, this research provides a comprehensive overview of what patient-reported measures can be used by healthcare professionals to evaluate virtual care quality. As virtual care represents a burgeoning approach to care provision, utilizing these measures (PROMs/PREMs) can be crucial to ensuring that the services provided are grounded in patient-centeredness [129]. This study has implications on all conceivable aspects of virtual clinical practice, by equipping healthcare professionals with the means to respond to the needs of their specific patient population.

## Strengths and limitations

One key strength of this study was the patient-oriented approach. We engaged a patient research partner in our team who was involved in reviewing the study protocol, title and abstract screening, data abstraction, reviewing the results and is a co-author in this manuscript. Additionally, we enlisted the support of a research librarian to ensure our search strategy was comprehensive.

Despite the methodological rigor applied in this review, this study was not immune to limitations. One potential issue with this study is that, while the review included studies published between January 2020 and January 2022, the vast majority (90.2%) of included articles detailed work conducted in 2020. Limited information on patient experiences further into the pandemic restricted our ability to assess the effects of patient and family burnout from continued virtual care use. Another possible limitation of this study relates to our focus on patient and family responses to virtual care, exclusively. While this does exclude the perspectives of healthcare providers and administrators, our emphasis on the patient voice was also a deliberate choice to display the experiences of virtual care recipients. Another limitation in this study was our inability to perform a meta-analysis due to the inclusion of studies that differed across statistical and methodological characteristics. Lastly, though not a limitation of this review, a recurrent issue experienced in studies examining the use of PREMs, is the common, yet misguided practice, of using “experience” and “satisfaction” as interchangeable terms. These terms, while seemingly similar, do have distinct qualities with “satisfaction” associated with greater subjectivity and potentially reflecting patient expectations more so than “experience” which describes objective aspects of patient care [130, 131].

## Conclusions

In future studies, it would be efficacious to explore more recent patient experiences with virtual care as well as the experiences of other key stakeholders. Improved patient receptivity to care at the onset of the pandemic has been previously documented, however, patient experiences further into the pandemic is lacking. Due to widespread burnout within the healthcare system, assessing more recently completed patient-reported measures may paint a different picture of the benefits of virtual care [132]. Additionally, further research into healthcare professionals' perspectives (I.e., healthcare providers and administrators) would offer an alternative lens on the practicality and feasibility of long-term virtual care.

### Supplementary Information

**Additional file 1.** Search Strategy.

**Additional file 2.** Quality Assessment of Included Studies.

## Data Availability

Data available upon request to the authors.
