# Supplementary material for: Patient-reported experiences and outcomes of virtual care during COVID-19: a systematic review
Source: J Patient Rep Outcomes. 2023 Dec 1;7:126. doi: 10.1186/s41687-023-00659-8 (PMC10692047; doi:10.1186/s41687-023-00659-8)
Supplement: Supplementary file 1 — Additional file 1. Search Strategy. [file 41687_2023_659_MOESM1_ESM.docx]

**Search Keywords: Delivery of virtual care during COVID-19**

**Appendix A – Search terms (search date on January 29, 2022)**

| Database | Number of References |
| --- | --- |
| MEDLINE | 1926 |
| CINAHL | 2423 |
| Embase | 2261 |
| APA PsycInfo | 576 |
| Other sources | 1 |
| TOTAL | **7187** |
| Duplicate | 1139 |

**MEDLINE search terms (1926 studies)**

1. COVID-19/ or exp COVID-19 Testing/ or COVID-19 Vaccines/ or SARS-CoV-2/
2. (coronavirus/ or betacoronavirus/ or coronavirus infections/) and (disease outbreaks/ or epidemics/ or pandemics/)
3. (nCoV* or 2019nCoV or 19nCoV or COVID19* or COVID or SARS-COV-2 or SARSCOV-2 or SARS-COV2 or SARSCOV2 or SARS coronavirus 2 or Severe Acute Respiratory Syndrome Coronavirus 2 or Severe Acute Respiratory Syndrome Corona Virus 2).ti,ab,kf.
4. ((new or novel or Wuhan or Hubei or China or Chinese) adj3 (coronavirus* or corona virus* or betacoronavirus* or CoV or HCoV)).ti,ab,kf.
5. (longCOVID* or postCOVID* or postcoronavirus* or postSARS*).ti,ab,kf.
6. ((coronavirus* or corona virus* or betacoronavirus*) adj3 (pandemic* or epidemic* or outbreak* or crisis)).ti,ab,kf.
7. ((Wuhan or Hubei) adj5 pneumonia).ti,ab,kf.
8. 1 or 2 or 3 or 4 or 5 or 6 or 7
9. exp Remote Consultation/ or exp Telemedicine/ or Distance Counseling/
10. ((comput* or distance or internet or phone or online or remote or digital or tele* or video or virtual or web) adj2 (administ* or advice or assess* or care or chat* or confer* or consult* or counsel* or deliver* or health* or interv* or manag* or medic* or monit* or nurs* or pharm* or therap* or visit*)).ti,ab,kf.
11. (teleadminist* or teleadvice or teleassess* or telecare or telechat* or teleconf* or teleconsult* or telecounsel* or teledeliv* or telehealth* or teleinterv* or telemanag* or telemedic* or telemonit* or telenurs* or telepharm* or televisit* or teletherap* or videochat* or videocunsult* or videocunsel* or videotelephon*).ti,ab,kf.
12. (eConsult* or e-consult* or ecounsel* or e-counsel* or eHealth* or e-Health* or einterv* or e-interv* or etherap* or e-therap* or mHealth* or m-Health* or mobile health*).ti,ab,kf.
13. Telemed*.ti,ab,kf.
14. ((comput* or distance or electronic or internet or phone or smartphone or online or remote or tele* or video or virtual or web) adj2 (derm* or neurol* or pathol* or psych* or radiol* or rehab* or surg*)).ti,ab,kf.
15. (telederm* or teleneurol* or telepathol* or telepsych* or teleradiol* or telerehab* or telesurg* or telecare* or tele-care*).ti,ab,kf.
16. (App or apps or facetime* or helpline* or store-and-forward* or store-forward* or skype* or video* or zoom or webbased tool or web-based tool* or voice-over or voiceover or VoIP).ti,ab,kf.
17. exp Delivery of Health Care/ or (health care delivery or care delivery).ti,ab,kf.
18. 9 or 10 or 11 or 12 or 13 or 14 or 15 or 16 or 17
19. exp Patient Satisfaction/ or (satisfaction* or patient experience*).ti,ab,kf.
20. (patient-reported outcome* or Patient Reported Outcome Measure*).ti,ab,kf.
21. patient outcome*.ti,ab,kf.
22. patient-reported experience measure*.ti,ab,kf.
23. (caregiver experience* or family experience*).ti,ab,kf.
24. (health outcome* or health care outcome*).ti,ab,kf.
25. (health utilization* or utilisation* or health care utilization* or utilisation* or health services utilization* or utilisation* or health care service utilization* or utilisation*).ti,ab,kf.
26. (Emergency Medical Service* or Emergency department visit*).ti,ab,kf.
27. (hospitalization or hospital readmission or Patient Readmission).ti,ab,kf.
28. (physician visit* or general practitioner visit*).ti,ab,kf.
29. 19 or 20 or 21 or 22 or 23 or 24 or 25 or 26 or 26 or 27 or 28
30. 8 and 18 and 29
31. Limit 30 to (English language and humans and yr=”2020 -Current”)

**CINAHL search terms (2423 studies)**

1. (MH "COVID-19 Pandemic") OR (MH "COVID-19") OR (MH "SARS-CoV-2") OR (MH "Coronavirus Infections+") OR (MH "Coronavirus+")
2. 2019-nCoV* or 2019nCov* or coronavirus* or coronavirus 2 or coronavirus2* or corona or covid or covid-19 or covid19* or novel coronavirus* or SARS-COV-2* or SARS-COV2* or SARSCoV-2* or SARSCoV2* or Severe Acute Respiratory Syndrome Coronavirus 2 or Severe Acute Respiratory Syndrome Corona Virus 2 or (Wuhan N3 (pneumonia or virus))
3. 229E or HCov* or HKU1 or NL63 or OC43 or ncov* or sarscov*
4. 1 or 2 or 3
5. (MH "Telenursing") OR (MH "Telehealth+") OR (MH "Telemedicine+") OR (MH "Telerehabilitation") OR (MH "Telepsychiatry")
6. (MH "Videorecording+") OR (MH "Remote Consultation") OR (MH "Telephone Consultation (Iowa NIC)")
7. teleadminist* or teleadvice or teleassess* or telecare or telechat* or teleconf* or teleconsult* or telecounsel* or teledeliv* or telehealth* or teleinterv* or telemanag* or telemedic* or telemonit* or telenurs* or telepharm* or televisit* or teletherap* or videochat* or videocunsult* or videocunsel* or videotelephon* or telederm* or teleneurol* or telepathol* or telepsych* or teleradiol* or telerehab* or telesurg* or telecare* or tele-care*
8. ( eConsult* or e-consult* or ecounsel* or e-counsel* or eHealth* or e-Health* or einterv* or e-interv* or etherap* or e-therap* or mHealth* or m-Health* or mobile health* ) OR ( comput* or distance or electronic or internet or phone or smartphone or online or remote or tele* or video or virtual or web ) OR ( App or apps or facetime* or helpline* or store-and-forward* or store-forward* or skype* or video* or zoom or webbased tool or web-based tool* or voice-over or voiceover or VoIP )
9. “Delivery of Health Care” or health care delivery or care delivery*
10. 5 or 6 or 7 or 8 or 9
11. "Patient Satisfaction OR Patient Preference OR Patient Experience OR Patient Opinions OR Patient View OR Patient Perspective"
12. (MH "Patient-Reported Outcomes+") OR (MH "Outcome Assessment") OR (MH "Outcomes (Health Care)+")
13. ( Patient Satisfaction or satisfaction* or patient experience* or patient-reported outcome* or Patient Reported Outcome Measure* or patient outcome* or patient-reported experience measure* or patient preference* or patient opinion* or patient perspective* ) OR ( caregiver experience* or family experience* )
14. health outcome* or health care outcome* or health utilization* or utilisation* or health care utilization* or utilisation* or health services utilization* or utilisation* or health care service utilization* or utilisation*
15. Emergency Medical Service* or Emergency department visit* or hospitalization* or hospital readmission* or Patient Readmission* or physician visit* or general practitioner visit*
16. 11 OR 12 OR 13 OR 14 OR 15
17. 4 AND 10 AND 16
18. **Limiters** - Published Date: 20200101-20220131; Scholarly (Peer Reviewed) Journals; English Language; Human

**Embase search terms (2261 studies)**

1. exp coronavirus disease 2019/
2. exp Coronaviridae infection/ or Coronaviridae/
3. exp severe acute respiratory syndrome/
4. (nCoV* or 2019nCoV or 19nCoV or COVID19* or COVID or SARS-COV-2 or SARSCOV-2 or SARS-COV2 or SARSCOV2 or SARS coronavirus 2 or Severe Acute Respiratory Syndrome Coronavirus 2 or Severe Acute Respiratory Syndrome Corona Virus 2).tw,kw.
5. (229E or HCov* or HKU1 or NL63 or OC43 or ncov* or sarscov*).tw,kw.
6. 1 or 2 or 3 or 4 or 5
7. exp telecommunication/ or exp telemedicine/ or exp teleconsultation/
8. ((comput* or distance or internet or phone or online or remote or digital or tele* or video or virtual or web) adj2 (administ* or advice or assess* or care or chat* or confer* or consult* or counsel* or deliver* or health* or interv* or manag* or medic* or monit* or nurs* or pharm* or therap* or visit*)).tw,kw.
9. (teleadminist* or teleadvice or teleassess* or telecare or telechat* or teleconf* or teleconsult* or telecounsel* or teledeliv* or telehealth* or teleinterv* or telemanag* or telemedic* or telemonit* or telenurs* or telepharm* or televisit* or teletherap* or videochat* or videocunsult* or videocunsel* or videotelephon*).tw,kw.
10. (eConsult* or e-consult* or ecounsel* or e-counsel* or eHealth* or e-Health* or einterv* or e-interv* or etherap* or e-therap* or mHealth* or m-Health* or mobile health*).tw,kw.
11. ((comput* or distance or electronic or internet or phone or smartphone or online or remote or tele* or video or virtual or web) adj2 (derm* or neurol* or pathol* or psych* or radiol* or rehab* or surg*)).tw,kw.
12. (telederm* or teleneurol* or telepathol* or telepsych* or teleradiol* or telerehab* or telesurg* or telecare* or tele-care*).tw,kw.
13. (App or apps or facetime* or helpline* or store-and-forward* or store-forward* or skype* or video* or zoom or webbased tool or web-based tool* or voice-over or voiceover or VoIP).tw,kw.
14. exp health care delivery/
15. 7 or 8 or 9 or 10 or 11 or 12 or 13 or 14
16. exp patient satisfaction/
17. exp patient-reported outcome/
18. (patient experience* or patient-reported outcome* or patient reported outcome* or patient outcome* or patient-reported experience measure* or patient perspective* or patient view* or caregiver experience* or family experience*).tw,kw.
19. exp health care utilization/
20. (health outcome* or health care outcome* or health care utilization* or health care utilisation* or emergency medical service* or emergency department visit* or hospital readmission or patient readmission or physician visit* or general practitioner visit*).tw,kw.
21. 16 or 17 or 18 or 19 or 20
22. 6 and 15 and 21
23. limit 22 to (human and english language and yr="2020 -Current" and article)

**APA PsycInfo search terms (576 studies)**

1. coronavirus.mp. or exp Coronavirus/
2. COVID-19.mp. or exp COVID-19/
3. (nCoV* or 2019nCoV or 19nCoV or COVID19* or COVID or SARS-COV-2 or SARSCOV-2 or SARS-COV2 or SARSCOV2 or SARS coronavirus 2 or Severe Acute Respiratory Syndrome Coronavirus 2 or Severe Acute Respiratory Syndrome Corona Virus 2).tw.
4. (coronavirus* or corona virus* or betacoronavirus* or pandemic* or epidemic* or outbreak* or crisis or new or novel).tw.
5. (229E or HCov* or HKU1 or NL63 or OC43 or ncov* or sarscov*).tw.
6. 1 or 2 or 3 or 4 or 5
7. exp Telemedicine/ or exp Online Therapy/ or exp Teleconferencing/ or exp Videoconferencing/
8. ((comput* or distance or internet or phone or online or remote or digital or tele* or video or virtual or web) adj2 (administ* or advice or assess* or care or chat* or confer* or consult* or counsel* or deliver* or health* or interv* or manag* or medic* or monit* or nurs* or pharm* or therap* or visit*)).tw.
9. (teleadminist* or teleadvice or teleassess* or telecare or telechat* or teleconf* or teleconsult* or telecounsel* or teledeliv* or telehealth* or teleinterv* or telemanag* or telemedic* or telemonit* or telenurs* or telepharm* or televisit* or teletherap* or videochat* or videocunsult* or videocunsel* or videotelephon*).tw.
10. (eConsult* or e-consult* or ecounsel* or e-counsel* or eHealth* or e-Health* or einterv* or e-interv* or etherap* or e-therap* or mHealth* or m-Health* or mobile health*).tw.
11. ((comput* or distance or electronic or internet or phone or smartphone or online or remote or tele* or video or virtual or web) adj2 (derm* or neurol* or pathol* or psych* or radiol* or rehab* or surg*)).tw.
12. (telederm* or teleneurol* or telepathol* or telepsych* or teleradiol* or telerehab* or telesurg* or telecare* or tele-care*).tw.
13. (App or apps or facetime* or helpline* or store-and-forward* or store-forward* or skype* or video* or zoom or webbased tool or web-based tool* or voice-over or voiceover or VoIP).tw.
14. exp Health Care Delivery/
15. 7 or 8 or 9 or 10 or 11 or 12 or 13 or 14
16. patient satisfaction.mp. or exp Client Satisfaction/
17. exp Treatment Outcomes/ or exp "Quality of Life"/ or exp Patient Reported Outcome Measures/ or exp Treatment Effectiveness Evaluation/
18. exp "Experiences (Events)"/ or patient experience.mp.
19. (patient satisfaction* or patient experience* or client experience* or consumer experience* or patient-reported outcome* or patient reported outcome* or patient outcome* or client outcome* or patient-reported experience measure* or patient perspective* or client perspective* or consumer perspective* or patient view* or client view* or consumer view* or caregiver experience* or family experience*).tw.
20. health care utilization.mp. or exp Health Care Utilization/
21. (health outcome* or health care outcome* or health care utilization* or health care utilisation* or emergency medical service* or emergency department visit* or hospital readmission or patient readmission or physician visit* or psychiatry visit* or general practitioner visit*).tw.
22. 16 or 17 or 18 or 19 or 20 or 21
23. 6 and 15 and 22
24. limit 23 to (peer reviewed journal and human and english language and yr="2020 -Current")

**Ovid MEDLINE(R)** and Epub Ahead of Print, In-Process, In-Data-Review & Other Non-Indexed Citations and Daily <1946 to January 27, 2022>

1 COVID-19/ or exp COVID-19 Testing/ or COVID-19 Vaccines/ or SARS-CoV-2/ 137253

2 (coronavirus/ or betacoronavirus/ or coronavirus infections/) and (disease outbreaks/ or epidemics/ or pandemics/) 40097

3 (nCoV* or 2019nCoV or 19nCoV or COVID19* or COVID or SARS-COV-2 or SARSCOV-2 or SARS-COV2 or SARSCOV2 or SARS coronavirus 2 or Severe Acute Respiratory Syndrome Coronavirus 2 or Severe Acute Respiratory Syndrome Corona Virus 2).ti,ab,kf. 211809

4 ((new or novel or Wuhan or Hubei or China or Chinese) adj3 (coronavirus* or corona virus* or betacoronavirus* or CoV or HCoV)).ti,ab,kf. 15886

5 (longCOVID* or postCOVID* or postcoronavirus* or postSARS*).ti,ab,kf. 26

6 ((coronavirus* or corona virus* or betacoronavirus*) adj3 (pandemic* or epidemic* or outbreak* or crisis)).ti,ab,kf. 11177

7 ((Wuhan or Hubei) adj5 pneumonia).ti,ab,kf,ot. 382

8 1 or 2 or 3 or 4 or 5 or 6 or 7 222881

9 exp Remote Consultation/ or exp Telemedicine/ or Distance Counseling/ 38903

10 ((comput* or distance or internet or phone or online or remote or digital or tele* or video or virtual or web) adj2 (administ* or advice or assess* or care or chat* or confer* or consult* or counsel* or deliver* or health* or interv* or manag* or medic* or monit* or nurs* or pharm* or therap* or visit*)).ti,ab,kf. 132486

11 (teleadminist* or teleadvice or teleassess* or telecare or telechat* or teleconf* or teleconsult* or telecounsel* or teledeliv* or telehealth* or teleinterv* or telemanag* or telemedic* or telemonit* or telenurs* or telepharm* or televisit* or teletherap* or videochat* or videocunsult* or videocunsel* or videotelephon*).ti,ab,kf. 32023

12 (eConsult* or e-consult* or ecounsel* or e-counsel* or eHealth* or e-Health* or einterv* or e-interv* or etherap* or e-therap* or mHealth* or m-Health* or mobile health*).ti,ab,kf. 20506

13 Telemed*.ti,ab,kf. 19852

14 ((comput* or distance or electronic or internet or phone or smartphone or online or remote or tele* or video or virtual or web) adj2 (derm* or neurol* or pathol* or psych* or radiol* or rehab* or surg*)).ti,ab,kf. 19648

15 (telederm* or teleneurol* or telepathol* or telepsych* or teleradiol* or telerehab* or telesurg* or telecare* or tele-care*).ti,ab,kf. 6692

16 (App or apps or facetime* or helpline* or store-and-forward* or store-forward* or skype* or video* or zoom or webbased tool or web-based tool* or voice-over or voiceover or VoIP).ti,ab,kf. 189110

17 exp Delivery of Health Care/ or (health care delivery or care delivery).ti,ab,kf. 1177665

18 9 or 10 or 11 or 12 or 13 or 14 or 15 or 16 or 17 1461820

19 exp Patient Satisfaction/ or (satisfaction* or patient experience*).ti,ab,kf. 232355

20 (patient-reported outcome* or Patient Reported Outcome Measure*).ti,ab,kf. 27912

21 patient outcome*.ti,ab,kf. 60468

22 patient-reported experience measure*.ti,ab,kf. 238

23 (caregiver experience* or family experience*).ti,ab,kf. 1581

24 (health outcome* or health care outcome*).ti,ab,kf. 66619

25 (health utilization* or utilisation* or health care utilization* or utilisation* or health services utilization* or utilisation* or health care service utilization* or utilisation*).ti,ab,kf. 30810

26 (Emergency Medical Service* or Emergency department visit*).ti,ab,kf. 19749

27 (hospitalization or hospital readmission or Patient Readmission).ti,ab,kf. 150481

28 (physician visit* or general practitioner visit*).ti,ab,kf. 3669

29 19 or 20 or 21 or 22 or 23 or 24 or 25 or 26 or 27 or 28 562302

30 8 and 18 and 29 2903

31 limit 30 to (english language and humans and yr="2020 -Current") 1926

**Embase <1974 to 2022 January 27>**

1 exp coronavirus disease 2019/ 180203

2 exp Coronaviridae infection/ or Coronaviridae/ 200797

3 exp severe acute respiratory syndrome/ 10379

4 (nCoV* or 2019nCoV or 19nCoV or COVID19* or COVID or SARS-COV-2 or SARSCOV-2 or SARS-COV2 or SARSCOV2 or SARS coronavirus 2 or Severe Acute Respiratory Syndrome Coronavirus 2 or Severe Acute Respiratory Syndrome Corona Virus 2).tw,kw. 215841

5 (229E or HCov* or HKU1 or NL63 or OC43 or ncov* or sarscov*).tw,kw. 6801

6 1 or 2 or 3 or 4 or 5 248957

7 exp telecommunication/ or exp telemedicine/ or exp teleconsultation/ 94076

8 ((comput* or distance or internet or phone or online or remote or digital or tele* or video or virtual or web) adj2 (administ* or advice or assess* or care or chat* or confer* or consult* or counsel* or deliver* or health* or interv* or manag* or medic* or monit* or nurs* or pharm* or therap* or visit*)).tw,kw. 184099

9 (teleadminist* or teleadvice or teleassess* or telecare or telechat* or teleconf* or teleconsult* or telecounsel* or teledeliv* or telehealth* or teleinterv* or telemanag* or telemedic* or telemonit* or telenurs* or telepharm* or televisit* or teletherap* or videochat* or videocunsult* or videocunsel* or videotelephon*).tw,kw. 41165

10 (eConsult* or e-consult* or ecounsel* or e-counsel* or eHealth* or e-Health* or einterv* or e-interv* or etherap* or e-therap* or mHealth* or m-Health* or mobile health*).tw,kw. 21696

11 ((comput* or distance or electronic or internet or phone or smartphone or online or remote or tele* or video or virtual or web) adj2 (derm* or neurol* or pathol* or psych* or radiol* or rehab* or surg*)).tw,kw. 31536

12 (telederm* or teleneurol* or telepathol* or telepsych* or teleradiol* or telerehab* or telesurg* or telecare* or tele-care*).tw,kw. 8469

13 (App or apps or facetime* or helpline* or store-and-forward* or store-forward* or skype* or video* or zoom or webbased tool or web-based tool* or voice-over or voiceover or VoIP).tw,kw. 266204

14 exp health care delivery/ 3715002

15 7 or 8 or 9 or 10 or 11 or 12 or 13 or 14 4078051

16 exp patient satisfaction/ 152884

17 exp patient-reported outcome/ 37359

18 (patient experience* or patient-reported outcome* or patient reported outcome* or patient outcome* or patient-reported experience measure* or patient perspective* or patient view* or caregiver experience* or family experience*).tw,kw. 183212

19 exp health care utilization/ 82007

20 (health outcome* or health care outcome* or health care utilization* or health care utilisation* or emergency medical service* or emergency department visit* or hospital readmission or patient readmission or physician visit* or general practitioner visit*).tw,kw. 137795

21 16 or 17 or 18 or 19 or 20 527494

22 6 and 15 and 21 4566

23 limit 22 to (human and english language and yr="2020 -Current" and article) 2261
